# Phylum barrier and *Escherichia coli* intra-species phylogeny drive the acquisition of antibiotic-resistance genes

**DOI:** 10.1099/mgen.0.000489

**Published:** 2021-08-26

**Authors:** Marie Petitjean, Bénédicte Condamine, Charles Burdet, Erick Denamur, Etienne Ruppé

**Affiliations:** ^1^​ IAME, INSERM, Université de Paris, F-75018 Paris, France; ^2^​ Département d’Epidémiologie, Biostatistique et Recherche Clinique, Hôpital Bichat, APHP, F-75018 Paris, France; ^3^​ Laboratoire de Génétique Moléculaire, Hôpital Bichat, APHP, F-75018 Paris, France; ^4^​ Laboratoire de Bactériologie, Hôpital Bichat, APHP, F-75018 Paris, France

**Keywords:** antibiotic-resistance genes, *Escherichia coli*

## Abstract

*
Escherichia coli* is a ubiquitous bacterium that has been widely exposed to antibiotics over the last 70 years. It has adapted by acquiring different antibiotic-resistance genes (ARGs), the census of which we aim to characterize here. To do so, we analysed 70 301 *E. coli* genomes obtained from the EnteroBase database and detected 1 027 651 ARGs using the AMRFinder, Mustard and ResfinderFG ARG databases. We observed a strong phylogroup and clonal lineage specific distribution of some ARGs, supporting the argument for epistasis between ARGs and the strain genetic background. However, each phylogroup had ARGs conferring a similar antibiotic class resistance pattern, indicating phenotypic adaptive convergence. The G+C content or the type of ARG was not associated with the frequency of the ARG in the database. In addition, we identified ARGs from anaerobic, non-* Proteobacteria* bacteria in four genomes of *E. coli *, supporting the hypothesis that the transfer between anaerobic bacteria and *E. coli* can spontaneously occur but remains exceptional. In conclusion, we showed that phylum barrier and intra-species phylogenetic history are major drivers of the acquisition of a resistome in *E. coli *.

## Data Summary

All the genomes are publicly available in the EnteroBase database (https://enterobase.warwick.ac.uk/species/index/ecoli).

**Impact Statement**

We analysed a large set of *Escherichia coli* genomes and searched for antibiotic-resistance genes (ARGs) using various databases. We observed that ARGs distributed according to the phylogenetic background of the strains, supporting the observation that constraints were at play within *E. coli *. Moreover, we identified four instances of putative transfers of ARGs from a phylum other than that of *E. coli *, stressing the strong inter-phyla barrier for ARG exchange. However, the capacity of the acquired ARGs to provide resistance against the most used antibiotic families did not differ according to the phylogenetic background, stressing that the different lineages of *E. coli* adapted to the antibiotic pressure with the acquisition of ARGs their genetic background could accommodate. This research is to our knowledge the first of its kind to study the acquired resistome of *E. coli *, an intestinal, ubiquitous bacterium that has been exposed to antibiotics from their earliest use. In this connection, the results could help in better understanding how bacteria adapt to antibiotics by acquiring ARGs.

## Introduction

*Escherichia coli* is a ubiquitous bacterium found in the intestinal microbiota of vertebrates. In the human gut microbiota, *E. coli* is the dominant species of the phylum *Proteobacteria* with a mean 10^8^ c.f.u. (g faeces)^–1^ [1, 2]. It is also commonly found in the digestive tract of mammals and birds, including livestock and poultry [3]. Hence, during the last 70 years, *E. coli* strains have been highly exposed to antibiotics used in human and animal health, as well as those used in agriculture. In response, *E. coli* has adapted through the acquisition of multiple genes encoding antibiotic resistance referred to as antibiotic-resistance genes (ARGs). As a consequence, acquired resistance in *E. coli* is linked to human activities [4].

In the pre-next generation sequencing (NGS) era, an exhaustive characterization of ARGs in a given species was challenged by the necessity to use as many PCRs as the genes targeted and perform experiments in a high number of isolates. With the development of NGS and genomics after 2005, the number of bacterial genomes made available has increased exponentially and the identification of ARGs was made easier via the use of *in silico* tools. Efforts have been made to collect and organize known ARG sequences in dedicated databases, with the first antibiotic resistance database (ARDB) released as early as 2008 [5, 6]. Since then, others such as ResFinder [6], CARD [7], ARG-ANNOT [8] and more recently AMRFinder [9] have followed. Typically, these databases include thousands of ARG nucleotide and/or amino-acid sequences previously identified in cultivable and/or pathogenic bacteria. However, their content is biased as they lack the ARGs found in bacteria that are difficult to culture and those of little relevance from a medical perspective, such as commensal, strict anaerobic bacteria from the gut microbiota. Nonetheless, we and others have found these bacteria do harbour a vast diversity of ARGs, and the latter actually differ from those found in the ARG databases [10, 11]. These ARGs have been made available in specific databases, namely FARMEDB [12], ResFinderFG [13] and Mustard [11], whose content slightly overlaps with that of conventional ARG databases (Fig. S1, available with the online version of this article). Indeed, very few observations support occurrence of a transfer of the ARG from intestinal commensals to *Proteobacteria* opportunistic pathogens such as *E. coli* [14]. Still, such transfer has proven to be possible. For instance, *tetX*, a gene encoding resistance to tetracyclines [15], was shown to be transferred from *Bacteroidetes* to *Proteobacteria *. Furthermore, some transfer events may have gone unseen due to the lack of genomic monitoring of a large number of strains and because of the lack of sampling.

While most of these ARGs are borne by mobile genetic elements such as integrons [16], plasmids [17, 18] or more rarely phages [19], some associations between their presence and specific *E. coli* phylogroups have been evidenced in the past based on phenotypic and genetic markers [20–23]. More recently, genomic data have confirmed such associations and extended them to more specific phylogenetic lineages [24, 25]. Some of these multidrug-resistant lineages are disseminating worldwide, such as clonal group A [sequence type (ST) 69 phylogroup D] [26] and more recently ST131 (phylogroup B2) [27]. In such clonal groups, strong associations have been evidenced between the within ST sub-clade, the plasmid type and the ARG content [28]. All these data argue for a complex cross-talk between the chromosomal background, the genetic support of the ARG and the ARG itself resulting from intergenic epistasis [29].

Since December 2015, EnteroBase [30], a public database including thousands of genomes from *E. coli */* Shigella* and other species (* Salmonella *, other *Escherichia* species, *Clostridioides *, *Vibrio *, *Yersinia *, *Helicobacter* and *Moraxella *), has been available. Beyond genomes, EnteroBase includes (with varying degrees of completeness) metadata linked to the strain itself (name, source, location, laboratory, species, serotype, disease and entry/update date) and to its sequencing process (N50, coverage). Still, no data regarding antibiotic resistance are available. In this study, we leveraged the high number of *E. coli* genomes in EnteroBase to characterize the acquired ARGs in *E. coli* and, more precisely, (i) to test for specific associations between the phylogenetic background of the strains and the presence of ARGs, and (ii) to evidence ARG transfer between non-* Proteobacteria* species and *E. coli* using metagenomic databases ResFinderFG [13] and Mustard [11].

## Methods

### Genomic database, species classification and *E. coli* phylogroup determination

A total of 82 063 available genomes was downloaded from *Escherichia/Shigella* EnteroBase (as of 1 February 2019). The genomes were classified according to their genera and species (* Shigella *, *Escherichia coli *, *Escherichia albertii *, *Escherichia fergusonii *, *Escherichia marmotae* or unknown). First, *Shigella* and enteroinvasive *E. coli* (EIEC) genomes were identified using *in silico* PCR with primers of the *ipaH3* gene [31]. Due to their specific, obligatory intracellular pathogenic trait, they were removed from the dataset. Then, using ClermonTyper [32], a tool that provides information about phylogroups (A, B1, B2, C, D, E, F and G) for *E. coli* and identifies nearest species in conjunction with Mash [33] (* E. fergusonii *, *E. albertii* and *Escherichia* clades including *E. marmotae *), all the genomes were classified as *E. coli* belonging to the aforementioned phylogroups, *E. fergusonii *, *E. albertii* and *Escherichia* clades I to V. Of note, in this study, we considered that phylogroup F included both F and G phylogroups [34]. When a discrepancy was observed between the ClermonTyper and Mash attribution (*n*=3734) the strain was classified according to Mash.

### ARG identification, plasmid incompatibility group, chromosomal multilocus sequence type (MLST) determination and G+C content

Diamond [35] was used to identify all the ARGs in EnteroBase by aligning all genomes against the AMRFinder [9] (29 04 2019 version), Mustard [11] (30 09 2017 version) and the ResFinderFG [13] (21 12 2016 version) databases (with a minimum coverage × identity value greater than 0.64 for nucleotidic sequences corresponding to 80 % identity and 80 % coverage). All redundancies (sequences sharing 100 % identity in nucleotides) between the databases were removed. The ARG families were selected according to the Mustard website (http://mgps.eu/Mustard). All ResFinderFG and Mustard ARGs originatng from non-* Proteobacteria* were further investigated. When a genome was found to include an ARG putatively originating from a non-* Proteobacteria *, contamination (i.e*.* the presence of multiple sequences of non-* Proteobacteria* along with that of *E. coli *) was assessed using Kraken [36]. PlasmidFinder [37] database together with Diamond [35] were used to determine the plasmid incompatibility groups found in each genome of the EnteroBase database (98 % identity and a minimum of 95 % coverage). MLST was determined using *mlst* software based on the Warwick University or Pasteur Institute MLST schemes available from the pubMLST database (https://github.com/tseemann/mlst) [38]. G+C content (%) deviation between *E. coli* and the acquired ARG was measured using the *E. coli* G+C defined previously [39]. ARG sub-family classification was obtained by clustering all the ARGs from the three databases (AMRFinder, Mustard and ResfinderFG) using cd-hit-est based on a 90 % identity threshold [40, 41].

### Statistical analysis and normalization

To circumvent the sequencing biases for the richness and diversity estimation, the data were normalized so that each phylogroup would include the same number of genomes (*n*=10 000) by re-sampling (for C, D and F phylogroups) or sub-sampling (for A, B1, B2 and E phylogroups) while maintaining the proportionality. The same protocol was applied to the STs. For all other statistical analysis, the complete dataset was used (without normalization). We tested the correlations between phylogroup, ST, plasmid incompatibility group and ARG using the *corrplot* package of R v3.4.2 and the *corrmat* function. The preferential distribution of some ARGs within phylogroups was tested using the Kruskal–Wallis test and Benjamini–Hochberg correction. The diversity of the ARGs in some STs was measured using the Shannon index in R (v3.4.2) with the *vegan* package. The number of distinct ARGs in phylogroups or STs was referred to as ARG richness. Logistic regression was performed using R (v3.4.2) and the *glm* function. We first tested all variables in a univariate model and afterwards included in the multivariate model all variables that had shown a *P* value <0.01. The *stepAIC* function of the mass package was used to performs stepwise model selection by Akaike information criterion (AIC).

## Results

### Distribution of the species and *E. coli* phylogroups among the *E. coli/Shigella* EnteroBase

**Fig. 1.**
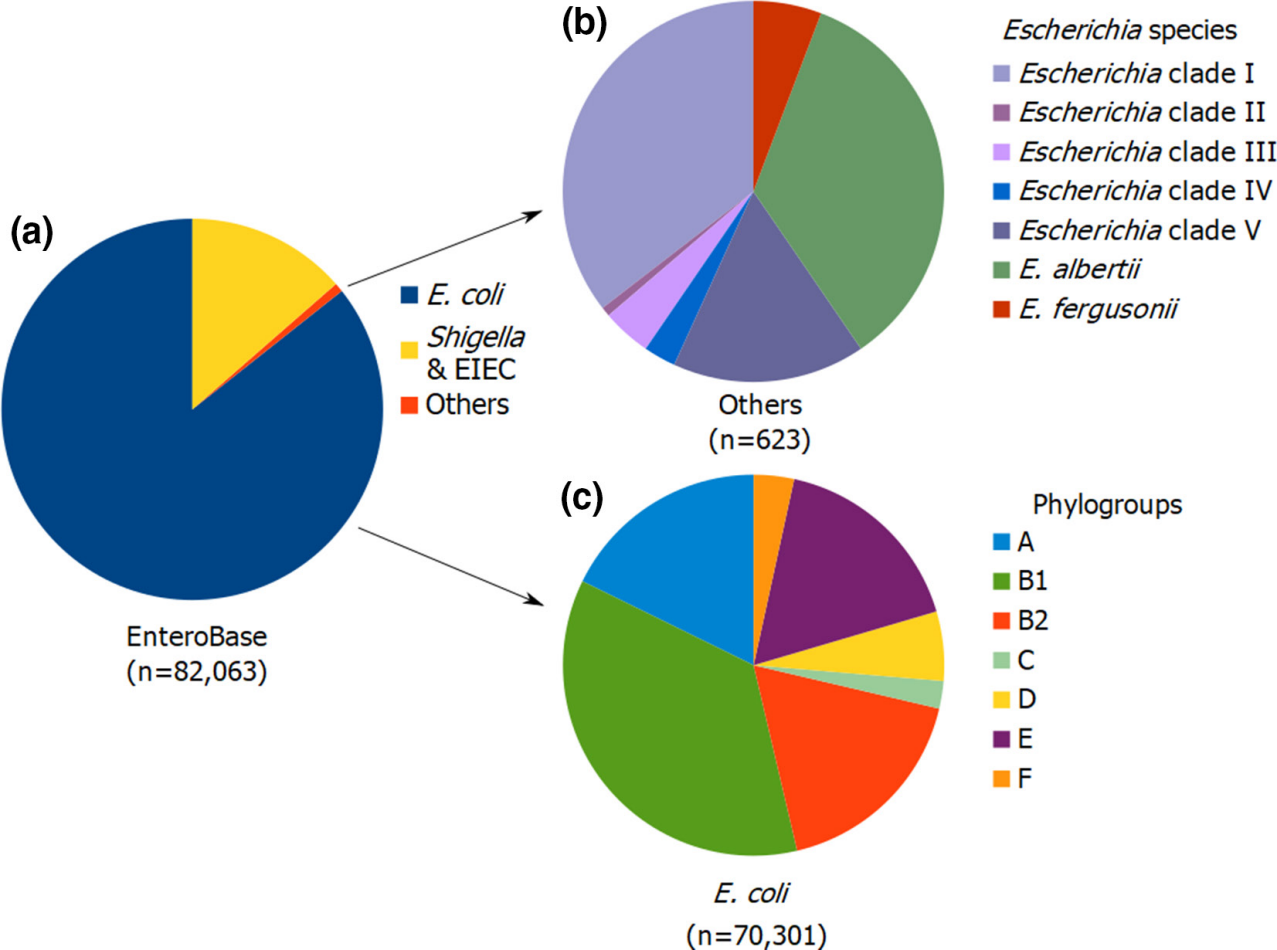
Distribution of species/phylogroups in the *Escherichia/Shigella* EnteroBase. Among the 82 063 genomes of the *Escherichia/Shigella* EnteroBase (**a**), we identified 623 genomes from *E. albertii *, *E. fergusonii* and *Escherichia* clades (referred to as ‘others’) (**b**), and 70 301 genomes of *E. coli* distributed in seven phylogroups (**c**). Of note, the F phylogroup in the figure includes both F and G phylogroups [34]. *Escherichia* clade III and IV are two sub-species belonging to a unique species. *Escherichia* clade V corresponds to *E. marmotae *.

During the curation step, 5144 genomes (6.3 % of genomes) were re-assigned to another genus/species. After curation, we identified 70 301 *E. coli *, 221 *Escherichia* clade I, 5 *Escherichia* clade II, 26 *Escherichia* clade III, 17 *Escherichia* clade IV, 102 *Escherichia* clade V (* E. marmotae *), 216 *E. albertii *, 36 *E. fergusonii* and 11 139 *Shigella* and EIEC (enteroinvasive *E. coli *). Among *E. coli *, the phylogroups were distributed as follows: B1 (25 265), A (12 469), B2 (12 414), E (11 965), D (4139), F (2403) and C (1646) (Fig. 1). The three major STs were ST11 (*n*=10 059, phylogroup E), ST131 (*n*=5143, phylogroup B2) and ST10 (*n*=3959, phylogroup A), making up 27 % of the *E. coli* in the database. Of note, ST11 and ST131 include the O157:H7 Shiga-toxin-producing *E. coli* (STEC) [42] and the O25b:H4 extra-intestinal pathogenic *E. coli* (ExPEC) strains [27], respectively, while ST10 encompasses the laboratory-derived K-12 strain [43].

### Most frequent ARGs found in *E. coli*

**Fig. 2.**
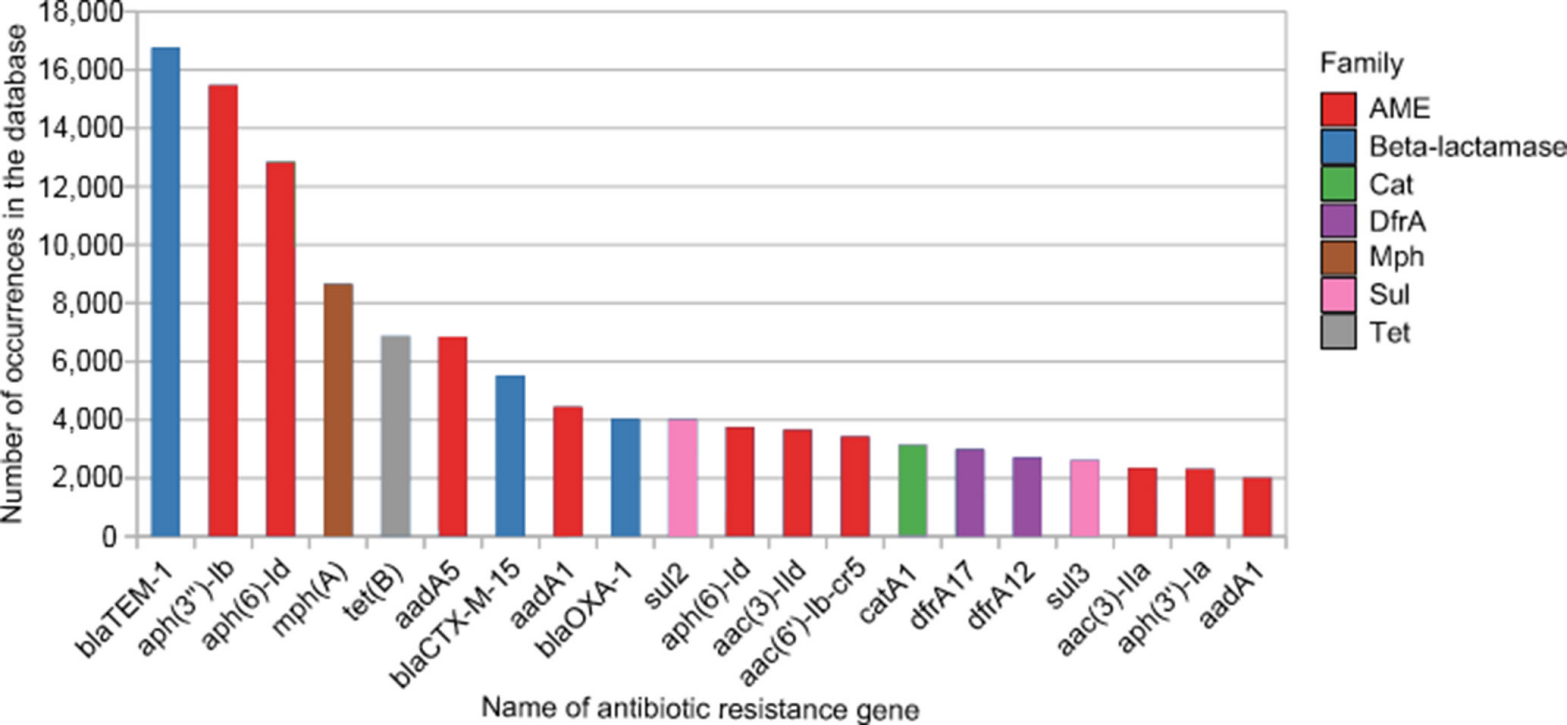
The 20 ARGs most frequently detected in *E. coli* from the EnteroBase database sharing 100 % identity with ARGs from AMRFinder. AME, aminoglycoside-modifying enzyme; Cat, chloramphenicol acetyltransferase; DfrA, dihydrofolate reductase type A; Mph, macrolide phosphotransferase; Sul, dihydropteroate synthase; Tet, tetracycline efflux pump.

First, we used the AMRFinder database and identified a total of 314 091 ARGs in *E. coli* genomes. The mean number of ARGs by genome was around 4.5 and the median was 2, and we observed a minimum of 0 ARGs and a maximum of 47 ARGs. The first part (*n*=164 519) included genes matching to known genes with 100 % of identity and coverage. This corresponded to 381 ARGs out of the 4955 genes included in the AMRFinder database. The second part (*n*=149 572) was made of variants sharing a coverage × identity value greater than 0.64 for nucleotide sequences (corresponding to 80 % identity and 80 % coverage). This comprised variants for 328 genes (including 169 genes not previously detected when the 100 % identity and coverage parameters applied) in the AMRFinder database. A total of 550 genes out of 4955 (11.1 %) of the AMRFinder database were, thus, found at least once in *E. coli* genomes. The 20 most frequent ARGs sharing 100 % identity with genes from AMRFinder are depicted in Fig. 2. We predominantly found genes encoding β-lactamases and aminoglycoside-modifying enzymes (AMEs), the three most abundant genes being *bla*_TEM-1_ (*n*=16 766), *aph(3′′)-Ib* (*n*=15 481) and *aph(6)-Id* (*n*=12 845).

Among the 234 β-lactamases found in the AMRFinder database, 27 sub-families were identified (with 100 % identity or in variants). The five β-lactamases *bla*_TEM_ (*n*=18 364), *bla*_CTX-M_ (*n*=10 640), *bla*_OXA-1-like_ (*n*=4075), *bla*_CMY-2-like_ (*n*=1994) and *bla*_SHV_ (*n*=518) were the most frequent (Table S1).

We also observed a high frequency of ARGs conferring resistance to antibiotics that are used to treat infections not caused by *E. coli* but rather caused by Gram-positive bacteria, such as rifampicin (*arr*, *n*=394/110, respectively, 100 % identity and variants) and macrolide-lincosamide [*lnu* (*n*=494/322), *mef* (*n*=258/21), *mphA* (*n*=8661/408), *erm* (*n*=81/1405), *vga (n*=1/0) and *msr* (*n*=76/2)].

Unexpectedly, we identified a *blaZ* gene commonly found in *Staphylococcus aureus* in an *E. coli* strain. However, subsequent analysis of the genome revealed that 10 % of reads were assigned to *S. aureus* and 90 % to *E. coli *. This 10 % of reads from *S. aureus* evenly distributed in the genome of *S. aureus* strain CFSAN007851, strongly supporting the hypothesis of a contamination prior to sequencing.

### Distribution of the resistance genes according to the strain phylogeny

**Fig. 3.**
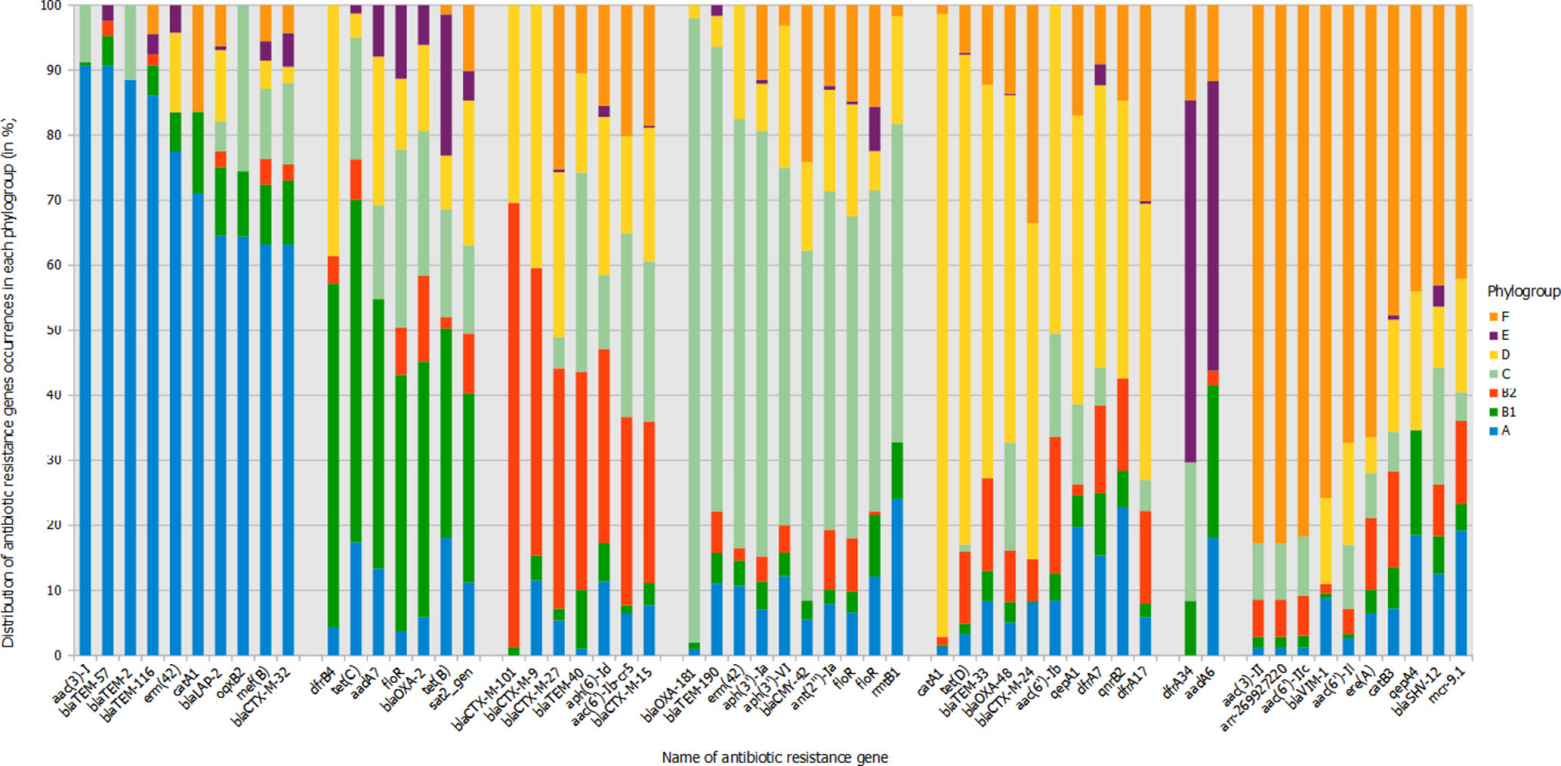
Histogram of the 10 (when available) most representative ARGs in each *E. coli* phylogroup (referred to as the PPRGs). Only genes with at least 20 occurrences in the database are represented. For each phylogroup, genes are sorted by decreasing frequency.

We observed that the mean number of ARGs per genome differs according to the phylogroup: 5.3 for A, 3.6 for B1, 5.5 for B2, 7.6 for C, 7.1 for D, 2.2 for E and 7.4 for F phylogroup (*P* <0.001 with ANOVA test). We took an ecological approach by considering the richness (corresponding to the number of unique ARGs) and the diversity with the Shannon index (used to quantify specific biodiversity). We observed a variable distribution of the ARG richness in each phylogroup, with a richness of 254, 236, 213, 170, 178, 122 and 169 in phylogroups A, B1, B2, C, D, E and F, respectively. However, we observed an even distribution of the diversity with Shannon index equal to 3.63, 3.51, 3.15, 3.58, 3.40, 2.92 and 3.51 (Fig. S2). The three most represented ARGs in each phylogroup were *bla*_TEM-1_, *aph(3′′)-Ib* and *aph(6)-Id*, except for in phylogroups D and F, in which *mphA* (a phosphotransferase conferring resistance to macrolides) and *tetB* (an efflux pump conferring resistance to tetracyclines) ranked second and third, respectively. However, specific ARGs were more frequently found in some phylogroups [referred to as phylogroup-predominant resistance genes (PPRGs), i.e. ARGs with a *P* value less than 0.001 with Kruskal–Wallis test and Benjamini–Hochberg correction; Fig. 3]. Indeed, 53.5 % (*n*=197/368) of the *bla*_OXA-48_ (a widely spread carbapenemase-encoding gene) were found in phylogroup D. Likewise, 49.5 % (*n*=4141/8366) of *bla*_CTX-M-15_ (encoding the extended-spectrum β-lactamase most prevalent worldwide) were found in phylogroups B2 and C (25 % each). Additionally, phylogroups B1 and E represented 25 265 and 11 965 genomes, but only included 5 and 35 PPRGs, respectively. In contrast, we observed 102 PPRGs in phylogroup A (*n*=12 469).

**Fig. 4.**
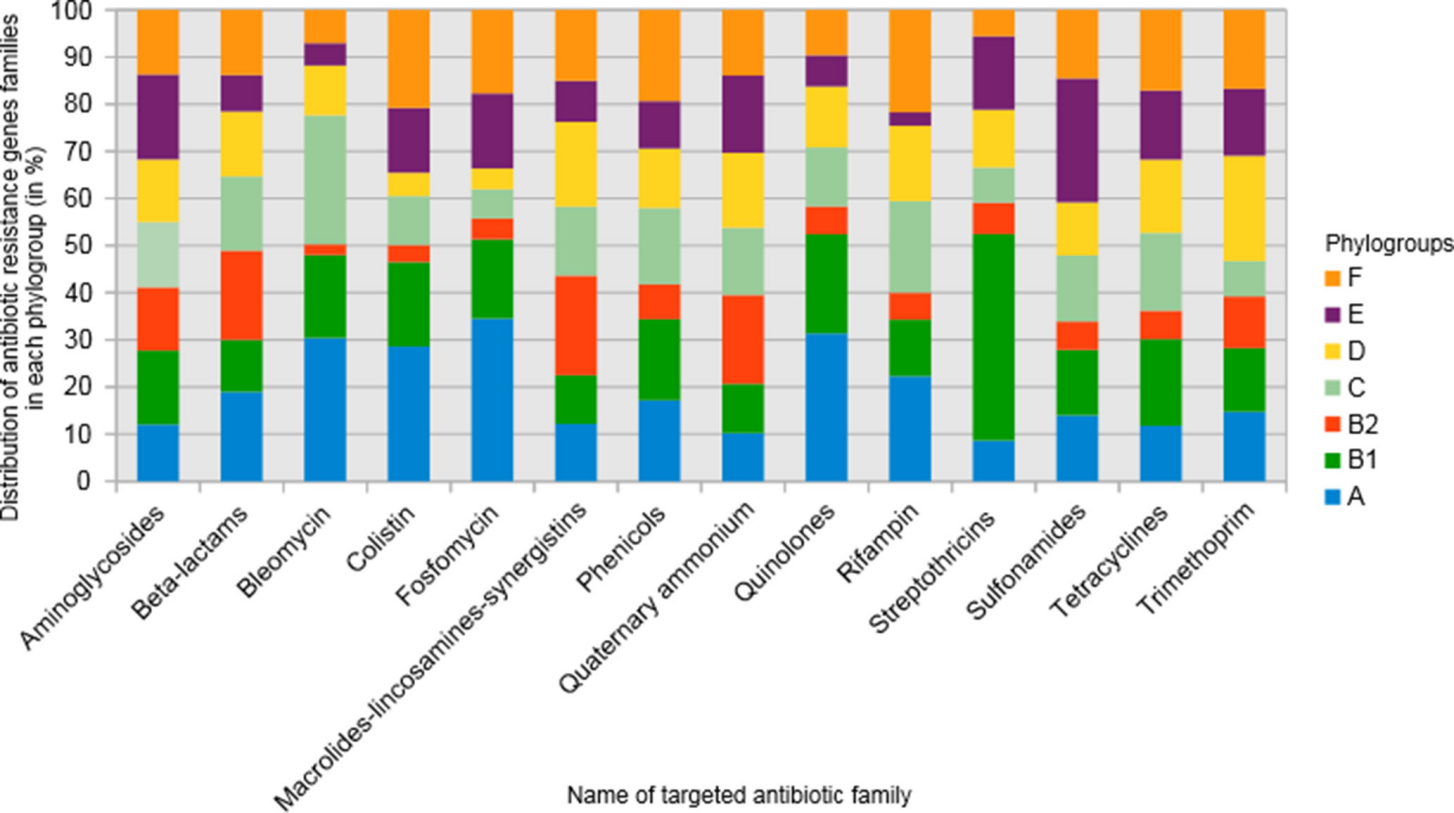
Histogram of the distribution of the targeted antibiotic families in each *E. coli* phylogroup. The proportion of each targeted antibiotic family is represented as a percentage of each phylogroup.

We tested the hypothesis that even if the distribution of ARGs differed according to the phylogroup, that of their function (i.e*.* the antibiotic families they encode resistance to) would not. We applied the same statistical approach and indeed found no specific association between the activity spectrum of ARG families and phylogroups (Fig. 4). For instance, we looked specifically at *bla*_CTX-M_ genes, which encode resistance to third-generation cephalosporins. *bla*_CTX-M_ was widespread between all phylogroups (and particularly in the phylogroup B2) except the phylogroup E where *bla*_CTX-M-1_ was prominent. However, the pattern of resistance they confer is very similar.

**Fig. 5.**
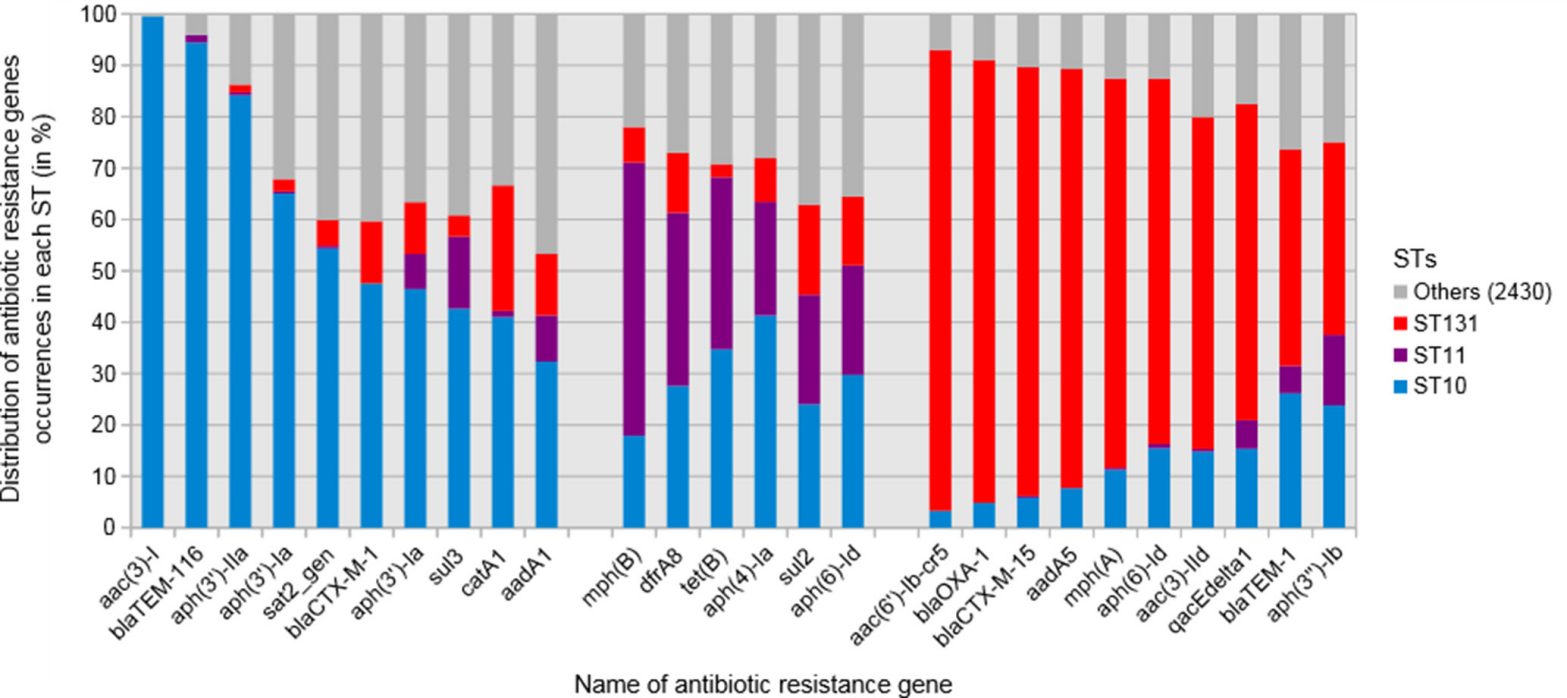
Histogram of the most representative ARGs in each *E. coli* major ST. Only genes with at least 20 occurrences in the database were represented. For each ST, genes were sorted by decreasing frequency. ‘Others’ represents the 2430 STs with few occurrences of the indicated genes. Each gene was preferentially found in one ST with a *P* value less than 0.01 with Kruskal–Wallis test and Benjamini–Hochberg correction.

We then searched for differential abundances with respect to the main STs of EnteroBase [ST11 (*n*=9391), ST131 (*n*=4841) and ST10 (*n*=3407)] (Fig. 5). Despite the fact that ST11 had the maximum number of genomes in EnteroBase, its ARG richness was 65, while it was 162 and 169 in ST131 and ST10, respectively. The Shannon entropy values for ST11, ST131 and ST10 were 2.36, 2.86 and 3.12, respectively. We also sought a specific distribution of some widely spread, clinically relevant β-lactamases. *bla*_TEM-1_ and *bla*_CTX-M-15_ were mostly found in ST131 [12.5 % (*n*=2094) and 42 % (*n*=2310)]. Conversely, *bla*_NDM-1_ was mostly found in ST101 (18 %, *n*=38).

### Global associations between strain phylogeny, plasmid type and resistance genes

We tested correlations between ARGs, phylogroups, STs and plasmid incompatibility groups. A total of 124 clusters were found with a correlation factor strictly higher than 0.30, each containing at least one ARG with other ARGs, STs, phylogroups or plasmid incompatibility groups. The *bla*_CTX-M-15_ gene strongly correlated with *aac(6′)-Ib* and *bla*_OXA-1_ (*r*=0.70), and also to a lesser extent correlated to *aac(3)-IIa*, *mphA*, *aadA5*, *qacEdelta1* and ST131, as well as the plasmid incompatibility group IncFII (*r*=0.36). In contrast, we did not identify other ARGs, plasmid incompatibility groups or STs associated with the other common *bla*_CTX-M_ genes, *bla*_CTX-M-27_ and *bla*_CTX-M-1_. As for carbapenemase-encoding genes, a correlation was detected between *bla*_NDM-1_ and *aph(3′)-VI*, *floR*, *erm-42*, *bla*_CMY-6_, *mphE*, *msrE*, *armA* and with the plasmid incompatibility group IncA (*r*=0.36). Finally, *bla*_TEM-1_ correlated with *aph(3′′)-Ib*, *aph(6)-Id*, *aac(3)-IId*, *sul2* and the incompatibility group IncQ1 (r=0.39). We further assessed whether the ARGs that correlated were located next to each other. The ARGs associated with *bla*_CTX-M-15_, *bla*_NDM-1_ and *bla*_TEM-1_ were in most instances found on the same contig (Fig. S3), supporting their acquisition via a common mobile genetic element rather than multiple acquisitions events. Last, we used a logistic regression to identify the variables associated with the presence of *bla*_CTX-M-15_, *bla*_NDM-1_ and *bla*_TEM-1_. In multivariate analysis, we observed strong associations between these genes and other ARGs (including those mentioned above), but also with plasmid types and phylogroups, supporting the role of the genetic background in the presence of ARGs in *E. coli* (Tables S2–S4). Of note, we did not observe any negative correlation between ARGs. However, genes *tetB*, *aadA5*, *qacEdelta1*, *bla_TEM-1_*, *bla_CTX-M-15_*, *bla_OXA-1_* and *mphA* were negatively correlated to ST11.

### G+C content and type of antibiotic resistance

**Fig. 6.**
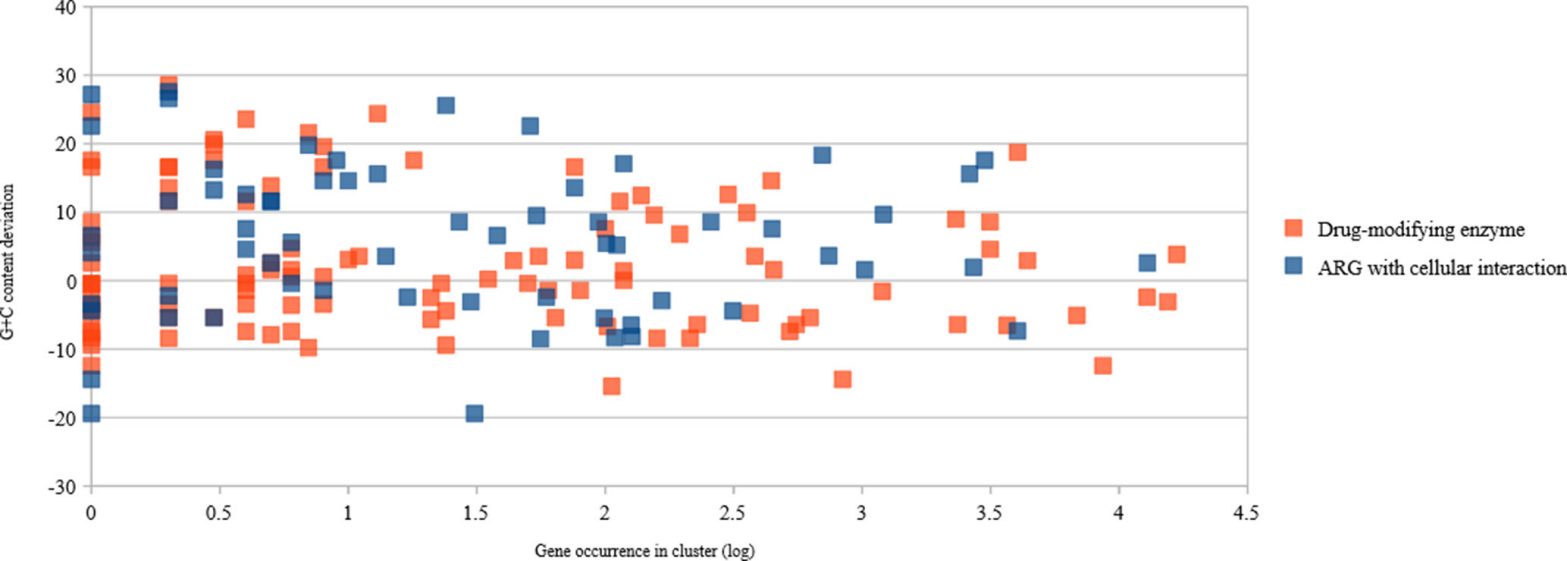
Scatter plot of the G+C deviation of ARGs according to their occurrence in EnteroBase and the type of resistance encoded. Each dot corresponds to one cluster of ARGs (90 % nucleotide identity) created using AMRFinder, Mustard and ResfinderFG databases. The G+C content deviation corresponds to the difference between the mean G+C content of *E. coli* [39] and the mean of the G+C content of each gene in the cluster.

We determined the G+C content of clusters of ARGs measured from the EnteroBase genomes and the distribution of their divergence using the mean G+C content of the *E. coli* core genome (51.8 %) [39]. We observed a large panel of G+C content deviation (Fig. 6) between the mean G+C content of *E. coli* and the G+C content of acquired ARGs, supporting the supposition that the G+C content was not a constraint for the acquisition of ARGs from other species or within the *E. coli* species. Interestingly, we also observed that the G+C content of the most frequent ARGs found in *E. coli* (with at least 1000 occurrences) did not significantly differ from that of the ARGs with low frequency (Mann–Whitney test, *P*=0.7, mean for the most frequent ARG 50.0, and 48.6 for low frequency).

The functional class of the horizontally transferred ARGs has been shown to play a role in the fitness of the recipient strain in that the gene encoding drug modification (e.g. β-lactamase and AME) would have a minimal impact on the fitness of the recipient strain even if originating from a G+C-divergent background. Conversely, genes encoding proteins interacting with the cellular content (e.g. efflux and target modification) would impact the fitness of the recipient, especially when originating from a G+C-divergent background [44]. However, in our dataset, we did not observe a distinct pattern according to the type of resistance (Mann–Whitney test, *P*=0.7, mean for drug-modifying enzyme equal to 3.969, and 2.582 for ARGs with cellular interactions; Fig. 6).

### Non-* Proteobacteria* ARGs can be exceptionally found in *E. coli*

Lastly, we assessed whether some ARGs found in non-* Proteobacteria* bacteria were present in the *E. coli* genomes of EnteroBase using the specific databases ResFinderFG and Mustard. We observed a high number of hits (*n*=833 814) in the Mustard and ResFinderFG databases, mostly corresponding to genes predicted to originate from *Proteobacteria *. The first part (*n*=271 458) included genes matching to known genes with 100 % of identity and coverage. This corresponded to 37 different genes identified in the 6095 genes of the Mustard database (Table S5) and 44 different genes identified in the 2282 genes of the ResFinderFG database (Table S6). The second part (*n*=562 356) was made of variants sharing a coverage × identity value greater than 0.64 for nucleotide sequences (corresponding to 80 % identity and 80 % coverage) with known genes. This made up 385 673 variants for 23 additional genes in Mustard (Table S5) and 176 683 variants for 61 additional genes in ResFinderFG (Table S6).

Interestingly, we identified a putative β-lactamase encoding gene from ResFinderFG (beta_lactamase, KU546399.1, faeces, AMX) and Mustard (MC3.MG60.AS1.GP1.C4251.G1) databases also found in the strain *Bacteroides uniformis* NBRC 113350 (NCBI GenBank accession no. NZ_AP019724.1). The *E. coli* bearing this gene was from phylogroup A, ST744 (Table 1), and had been isolated in Germany [45] from a patient screened for multidrug-resistant bacteria at the University Hospital of Münster. The gene was embedded in a 7600 bp contig (Fig. S4), itself sharing 100 % identity with the *Bacteroides uniformis* genome. The strain was kindly provided by Professor Alexander Mellmann from the University of Münster and was re-sequenced in our laboratory using Illumina MiniSeq and Oxford Nanopore (Oxford Nanopore Technologies) chemistries, which confirmed the presence of the resistance gene (data not shown). Further description of this strain is underway and will be detailed in a separate study.

**Table 1.** Description of the ARGs shared between *E. coli* and non-* Proteobacteria* strains

* E. coli* strain ID	Phylogroup	ST (Warwick University/Pasteur Institute schemes)	Serotype	*fimH* allele	Resistance gene	Strain of origin ID
ESC_HA9845AA	A	744/2	Onovel32:H10	54	Putative β-lactamase	* Bacteroides uniformis* strain NBRC 113350
ESC_OA1280AA	E	753/920	O130:H9	124	*erm*(49)	* Bifidobacterium breve* strain CECT7263
ESC_JA0734AA	D	405/477	O102:H6	27	*erm*	* Clostridioides difficile* strain CDT4
ESC_FA9928AA	A	5943/999	O89:H11	41	*tetM*	* Clostridioides difficile* strain CD161

We also found two *erm* genes (ribosomal methylases), encoding macrolide-lincosamide resistance and originating from non-* Proteobacteria *. The first one was found in an *E. coli* from phylogroup E, ST753, originating from a livestock sample from Ireland (Table 1). A part of the 12 910 bp contig containing the gene matched against a *Bifidobacterium breve* genome (Fig. S5). We were able to identify some transposase genes in the contig bearing the *erm* gene and surrounding inverted repeat sequences. The second *erm* was found in an *E. coli* from a phylogroup D, ST405 sample from The Netherlands (Table 1), and was originally identified on a plasmid from *Clostridioides difficile* (Fig. S6). Unfortunately, the metadata associated with those genomes was not sufficient to trace back the strains and confirm the presence of the genes in *E. coli* by re-sequencing the strains. However, no evidence of wet-lab or dry-lab contamination was observed.

Last, we identified a *tetM* gene, encoding tetracycline resistance, originating from *C. difficile* (Fig. S7). The gene was found in a 3291 bp contig completely matched against the *C. difficile* strain in an *E. coli* from phylogroup A, ST5943, originating from Thailand. No evidence of contamination was observed even though the reads were not available.

## Discussion

Using a large number of genomes, we were able to assess the diversity and distribution of ARGs in *E. coli *. From a global perspective, we assumed that the richness of acquired ARGs in *E. coli* was somewhat limited with regards to the high number of ARGs in the literature and the closeness of *E. coli* to human activities (the ARGs found in *E. coli* representing 11.1 % of the AMRFinder database). This suggested constraints in the path for ARG exchanges and sustainability between species (phylogenetic origin of the gene, interaction within the cell of the gene product [44, 46]), but also within the *E. coli* species. We did not find any evidence of a link between the G+C content or the functional class of the transferred genes and their frequency in the database. However, some ARGs had specific associations with the genomic background (phylogroups and STs), other ARGs and plasmid incompatibility groups. Of note, we did not observe any negative correlation between ARGs, suggesting that the ARGs are not competing with each other.

Such association between the phylogenetic background and the ARGs can result mainly from two evolutionary scenarios. In the ‘chance and timing scenario’, there is a limited number of acquisition events then propagated vertically (clonal inheritance). In this case, the strong gene-lineage association is only contingent upon evolutionary history. Such a rare acquisition scenario would be likely to apply to genes such as *aac(3)-I*, *aac(6′)-Ib-cr5* or *bla_TEM-116_*. Conversely, for the multiple arrivals, the maintenance and the expression of the ARGs are under selection due to epistatic interactions between the resistance determinants and the genomic background. This could concern genes such as *tetB*, with widespread distribution but increased prevalence in specific lineages.

Similar association between the genomic background and the presence of virulence genes has been reported within the *E. coli* species [47, 48] and attributed to epistasis between different parts of the genome [29]. Nonetheless, on a broader scale, we observed that these preferential genetic supports of resistance led to the same functional pattern of resistance to antibiotic classes. While *E. coli* phylogroups had different ARG distributions, they harboured a full genetic armamentarium to resist the same antibiotic families. In brief, the ARGs were different but their functions were similar. Such a functional redundancy suggests that the *E. coli* phylogroups were exposed to the same antibiotic pressure but acquired different ARGs to cope with it in an adaptive convergence process. Interestingly, a similar role of the genetic background influencing the genomic basis of antibiotic resistance by channelling evolution along different mutational paths has been reported following a long-term *in vitro* evolution experiment with *E. coli* [49].

The antibiotic exposure impacts not only the bacteria causing infections, but also the bacteria residing in our microbiota. Indeed, we observed a high rate of ARGs conferring resistance to antibiotics that are used to treat infections not caused by *E. coli* or other Gram-negative bacteria but rather caused by Gram-positive bacteria, such as rifampicin and macrolide-lincosamides. Such antibiotics are excreted in the intestine at high concentrations, so that some bacteria with minimum inhibitory concentrations too high to be in the clinical spectrum of the antibiotic would be affected in the gut. In that respect, the high frequency of ARGs conferring resistance to those antibiotics is a strong signal stressing the impact of antibiotics on our microbiota.

We observed four putative transfers of ARGs between non-* Proteobacteria* and *E. coli *. In a previous work, we observed that the vast majority of ARGs found in the intestinal microbiota were very distinct from those found in cultivable bacteria (including *E. coli *) and that few arguments were found to support their mobility [11]. Taking advantage of the largest *E. coli* database to date, we could observe that ARGs could actually be exchanged between *E. coli* and intestinal commensals, such as was observed for *tetX* [50]. Even if anecdotal in this dataset, with four observations, the very fact that they were detected suggests that they are not that uncommon, but unlike *tetX* which has met with success, their spread is very limited to date as they were only found in one genome each. The donor bacteria were strict anaerobic bacteria (* Bacteroides uniformis *, *Bifidobacterium breve* and *C. difficile *) commonly found in the gut microbiota at high abundances [51] alongside *E. coli *. Considering that humans have been using antibiotics for more than 70 years, very favourable conditions have been met for ARG transfers between anaerobic bacteria and *E. coli *. That such transfers have been observed so rarely supports the hypothesis that anaerobic bacteria may indeed provide ARGs to *E. coli *, but that the contribution to the worldwide AMR issue seems to be minor. Indeed, the most successful ARGs found in *E. coli* originate from other *Proteobacteria* species [14], e.g*. bla*_CTX-M_ progenitors are *Kluyvera* spp. belonging to the *Enterobacterales* [52]. Besides, the observations of the ARG transfer would not have been possible if not for the use of multiple databases to cover the broadest range of ARGs. While databases such as AMRFinder may be found suitable to identify ARGs from clinically relevant bacteria, they may not be appropriate when it comes to looking for ARGs originating from other environments, such as the gut microbiota.

We acknowledge some limitations of the present study. Despite including a large number of genomes, EnteroBase suffers from inclusion biases in that strains of interest (e.g*.* the resistant and/or the pathogenic ones) are the most sequenced. Indeed, EnteroBase includes a large number of STEC (Shiga-toxin-producing *E. coli *), mainly the O157:H7 serotype, ExPEC (extra-intestinal pathogenic *E. coli *) with the emerging ST131 and many strains producing extended spectrum β-lactamases. This may have led to an overestimation of the associations between ARGs, phylogenetic traits and replicons. Also, many more ARGs may have been found in *E. coli *, perhaps including some from intestinal commensals that were not found of interest to be cultured and sequenced. Hence, we assume we did not cover the global picture of the acquired ARGs always found in *E. coli *, but only a part. Nonetheless, we believe our findings are sound with regards to the very high number of strains included in this study. We also know that some genomes with contamination can be found in the database, which warrants the use of specific tools upstream of genome analysis.

In all, we observed that ARGs were distributed in the *E. coli* phylogroups/STs with a preferential fashion. In the meantime, they provided resistance to the same antibiotic families. Furthermore, we observed that the transfer of ARGs between non-* Proteobacteria* and *E. coli* indeed occurred but seemed to be exceptional.

## Supplementary Data

Supplementary material 1

Supplementary material 2

## References

[1] Slanetz LW, Bartley CH (1957). Numbers of enterococci in water, sewage, and feces determined by the membrane filter technique with an improved medium. J Bacteriol.

[2] Tenaillon O, Skurnik D, Picard B, Denamur E (2010). The population genetics of commensal *Escherichia coli*. Nat Rev Microbiol.

[3] Smati M, Clermont O, Bleibtreu A, Fourreau F, David A (2015). Quantitative analysis of commensal *Escherichia coli* populations reveals host-specific enterotypes at the intra-species level. Microbiologyopen.

[4] Skurnik D, Ruimy R, Andremont A, Amorin C, Rouquet P (2006). Effect of human vicinity on antimicrobial resistance and integrons in animal faecal *Escherichia coli*. J Antimicrob Chemother.

[5] Liu B, Pop M (2009). ARDB – antibiotic resistance genes database. Nucleic Acids Res.

[6] Zankari E, Hasman H, Cosentino S, Vestergaard M, Rasmussen S (2012). Identification of acquired antimicrobial resistance genes. J Antimicrob Chemother.

[7] Jia B, Raphenya AR, Alcock B, Waglechner N, Guo P (2017). CARD 2017: expansion and model-centric curation of the comprehensive antibiotic resistance database. Nucleic Acids Res.

[8] Gupta SK, Padmanabhan BR, Diene SM, Lopez-Rojas R, Kempf M (2014). ARG-ANNOT, a new bioinformatic tool to discover antibiotic resistance genes in bacterial genomes. Antimicrob Agents Chemother.

[9] Feldgarden M, Brover V, Haft DH, Prasad AB, Slotta DJ (2019). Using the NCBI AMRFinder tool to determine antimicrobial resistance genotype-phenotype correlations within a collection of narms isolates. bioRxiv.

[10] Sommer MOA, Dantas G, Church GM (2009). Functional characterization of the antibiotic resistance reservoir in the human microflora. Science.

[11] Ruppé E, Ghozlane A, Tap J, Pons N, Alvarez AS (2019). Prediction of the intestinal resistome by a three-dimensional structure-based method. Nat Microbiol.

[12] Wallace JC, Port JA, Smith MN, Faustman EM (2017). FARME DB: a functional antibiotic resistance element database. Database.

[13] Munk P, Knudsen BE, Lukjancenko O, Duarte ASR, Van Gompel L (2018). Abundance and diversity of the faecal resistome in slaughter pigs and broilers in nine European countries. Nat Microbiol.

[14] Ebmeyer S, Kristiansson E, Larsson DGJ (2021). A framework for identifying the recent origins of mobile antibiotic resistance genes. Commun Biol.

[15] Deng M, Zhu M-H, Li J-J, Bi S, Sheng Z-K (2014). Molecular epidemiology and mechanisms of tigecycline resistance in clinical isolates of *Acinetobacter baumannii* from a Chinese university hospital. Antimicrob Agents Chemother.

[16] Stalder T, Barraud O, Casellas M, Dagot C, Ploy MC (2012). Integron involvement in environmental spread of antibiotic resistance. Front Microbiol.

[17] Branger C, Ledda A, Billard-Pomares T, Doublet B, Barbe V (2019). Specialization of small non-conjugative plasmids in *Escherichia coli* according to their family types. Microb Genom.

[18] Branger C, Ledda A, Billard-Pomares T, Doublet B, Fouteau S (2018). Extended-spectrum β-lactamase-encoding genes are spreading on a wide range of *Escherichia coli* plasmids existing prior to the use of third-generation cephalosporins. Microb Genom.

[19] Billard-Pomares T, Fouteau S, Jacquet ME, Roche D, Barbe V (2014). Characterization of a P1-like bacteriophage carrying an SHV-2 extended-spectrum β-lactamase from an *Escherichia coli* strain. Antimicrob Agents Chemother.

[20] Branger C, Zamfir O, Geoffroy S, Laurans G, Arlet G (2005). Genetic background of *Escherichia coli* and extended-spectrum beta-lactamase type. Emerg Infect Dis.

[21] Deschamps C, Clermont O, Hipeaux MC, Arlet G, Denamur E (2009). Multiple acquisitions of CTX-M plasmids in the rare D2 genotype of *Escherichia coli* provide evidence for convergent evolution. Microbiology.

[22] Johnson JR, Goullet P, Picard B, Moseley SL, Roberts PL (1991). Association of carboxylesterase B electrophoretic pattern with presence and expression of urovirulence factor determinants and antimicrobial resistance among strains of *Escherichia coli* that cause urosepsis. Infect Immun.

[23] Johnson JR, Orskov I, Orskov F, Goullet P, Picard B (1994). O, K, and H antigens predict virulence factors, carboxylesterase B pattern, antimicrobial resistance, and host compromise among *Escherichia coli* strains causing urosepsis. J Infect Dis.

[24] Horesh G, Blackwell GA, Tonkin-Hill G, Corander J, Heinz E (2021). A comprehensive and high-quality collection of *Escherichia coli* genomes and their genes. Microb Genom.

[25] Touchon M, Perrin A, de Sousa JAM, Vangchhia B, Burn S (2020). Phylogenetic background and habitat drive the genetic diversification of *Escherichia coli*. PLoS Genet.

[26] Manges AR, Johnson JR, Foxman B, O’Bryan TT, Fullerton KE (2001). Widespread distribution of urinary tract infections caused by a multidrug-resistant *Escherichia coli* clonal group. N Engl J Med.

[27] Nicolas-Chanoine MH, Bertrand X, Madec JY (2014). *Escherichia coli* ST131, an intriguing clonal group. Clin Microbiol Rev.

[28] Kondratyeva K, Salmon-Divon M, Navon-Venezia S (2020). Meta-analysis of pandemic *Escherichia coli* ST131 plasmidome proves restricted plasmid-clade associations. Sci Rep.

[29] Domingo J, Baeza-Centurion P, Lehner B (2019). The causes and consequences of genetic interactions (epistasis. Annu Rev Genomics Hum Genet.

[30] Zhou Z, Alikhan N-F, Mohamed K, Fan Y, Agama Study Group (2020). The EnteroBase user’s guide, with case studies on *Salmonella* transmissions, *Yersinia pestis* phylogeny, and *Escherichia* core genomic diversity. Genome Res.

[31] Sahl JW, Morris CR, Emberger J, Fraser CM, Ochieng JB (2015). Defining the phylogenomics of *Shigella* species: a pathway to diagnostics. J Clin Microbiol.

[32] Beghain J, Bridier-Nahmias A, Le Nagard H, Denamur E, Clermont O (2018). ClermonTyping: an easy-to-use and accurate *in silico* method for *Escherichia* genus strain phylotyping. Microb Genom.

[33] Ondov BD, Treangen TJ, Melsted P, Mallonee AB, Bergman NH (2016). Mash: fast genome and metagenome distance estimation using MinHash. Genome Biol.

[34] Clermont O, Dixit OVA, Vangchhia B, Condamine B, Dion S (2019). Characterization and rapid identification of phylogroup G in *Escherichia coli,* a lineage with high virulence and antibiotic resistance potential. Environ Microbiol.

[35] Buchfink B, Xie C, Huson DH (2015). Fast and sensitive protein alignment using DIAMOND. Nat Methods.

[36] Wood DE, Lu J, Langmead B (2019). Improved metagenomic analysis with Kraken 2. Genome Biol.

[37] Carattoli A, Zankari E, García-Fernández A, Larsen MV, Lund O (2014). *In silico* detection and typing of plasmids using plasmidFinder and plasmid multilocus sequence typing. Antimicrob Agents Chemother.

[38] Jolley KA, Maiden MC (2010). BIGSdb: Scalable analysis of bacterial genome variation at the population level. BMC Bioinformatics.

[39] Bohlin J, Eldholm V, Pettersson JHO, Brynildsrud O, Snipen L (2017). The nucleotide composition of microbial genomes indicates differential patterns of selection on core and accessory genomes. BMC Genomics.

[40] Fu L, Niu B, Zhu Z, Wu S, Li W (2012). CD-HIT: accelerated for clustering the next-generation sequencing data. Bioinformatics.

[41] Li W, Godzik A (2006). Cd-hit: a fast program for clustering and comparing large sets of protein or nucleotide sequences. Bioinformatics.

[42] Kim S-W, Karns JS, Van Kessel JS, Haley BJ (2017). Genome sequences of 30 *Escherichia coli* O157:H7 isolates recovered from a single dairy farm and its associated off-site heifer-raising facility. Genome Announc.

[43] Blattner FR, Plunkett G, Bloch CA, Perna NT, Burland V (1997). The complete genome sequence of *Escherichia coli* K-12. Science.

[44] Porse A, Schou TS, Munck C, Ellabaan MMH, Sommer MOA (2018). Biochemical mechanisms determine the functional compatibility of heterologous genes. Nat Commun.

[45] Mellmann A, Bletz S, Böking T, Kipp F, Becker K (2016). Real-time genome sequencing of resistant bacteria provides precision infection control in an institutional setting. J Clin Microbiol.

[46] Jain R, Rivera MC, Lake JA (1999). Horizontal gene transfer among genomes: the complexity hypothesis. Proc Natl Acad Sci USA.

[47] Denamur E, Clermont O, Bonacorsi S, Gordon D (2021). The population genetics of pathogenic *Escherichia coli*. Nat Rev Microbiol.

[48] Escobar-Páramo P, Clermont O, Blanc-Potard AB, Bui H, Le Bouguénec C (2004). A specific genetic background is required for acquisition and expression of virulence factors in *Escherichia coli*. Mol Biol Evol.

[49] Card KJ, Thomas MD, Graves JL, Barrick JE, Lenski RE (2021). Genomic evolution of antibiotic resistance is contingent on genetic background following a long-term experiment with *Escherichia coli*. Proc Natl Acad Sci USA.

[50] Leski TA, Bangura U, Jimmy DH, Ansumana R, Lizewski SE (2013). Multidrug-resistant tet(X)-containing hospital isolates in Sierra Leone. Int J Antimicrob Agents.

[51] Li J, Jia H, Cai X, Zhong H, Feng Q (2014). An integrated catalog of reference genes in the human gut microbiome. Nat Biotechnol.

[52] Bonnet R (2004). Growing group of extended-spectrum beta-lactamases: the CTX-M enzymes. Antimicrob Agents Chemother.

