# Supplementary material for: Phylum barrier and Escherichia coli intra-species phylogeny drive the acquisition of antibiotic-resistance genes
Source: Microb Genom. 2021 Aug 26;7(8):000489. doi: 10.1099/mgen.0.000489 (PMC8549366; doi:10.1099/mgen.0.000489)
Supplement: Supplementary material 1 [file mgen-7-0489-s001.pdf]

Supplementary Figures and Tables

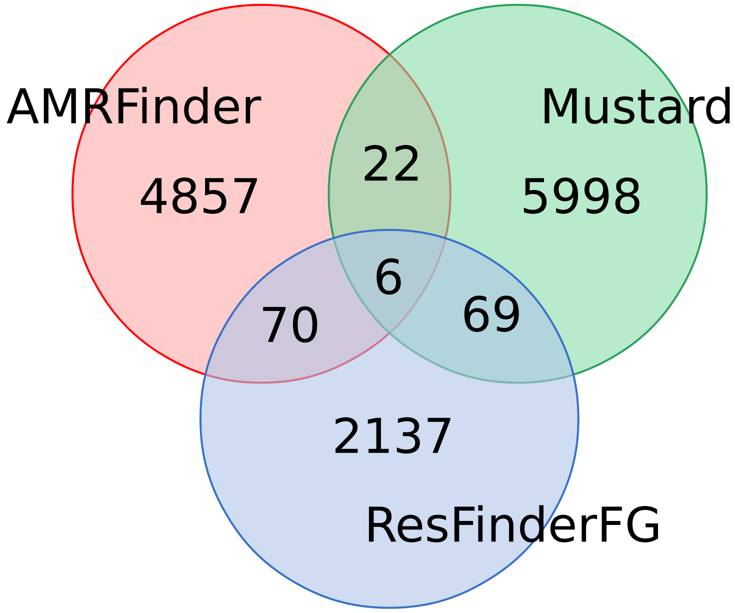

Supplementary Figure 1: Venn diagram for the three database used.

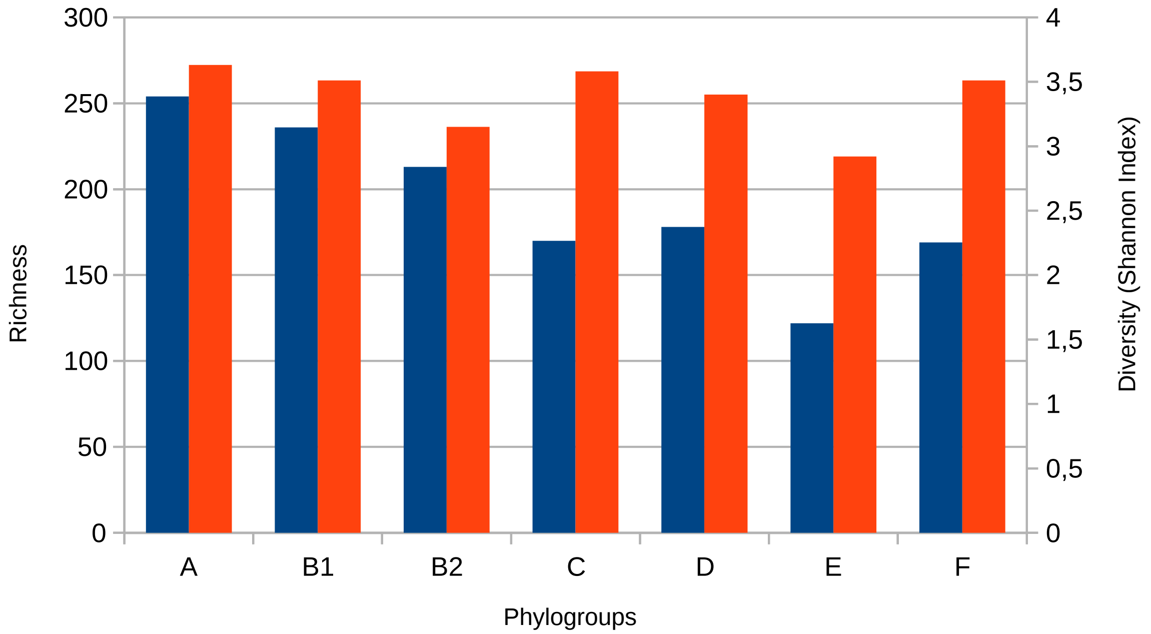

Supplementary Figure 2: Histogram of the richness and diversity for each phylogroup. Richness was represented in blue and diversity index (Shannon) in red.

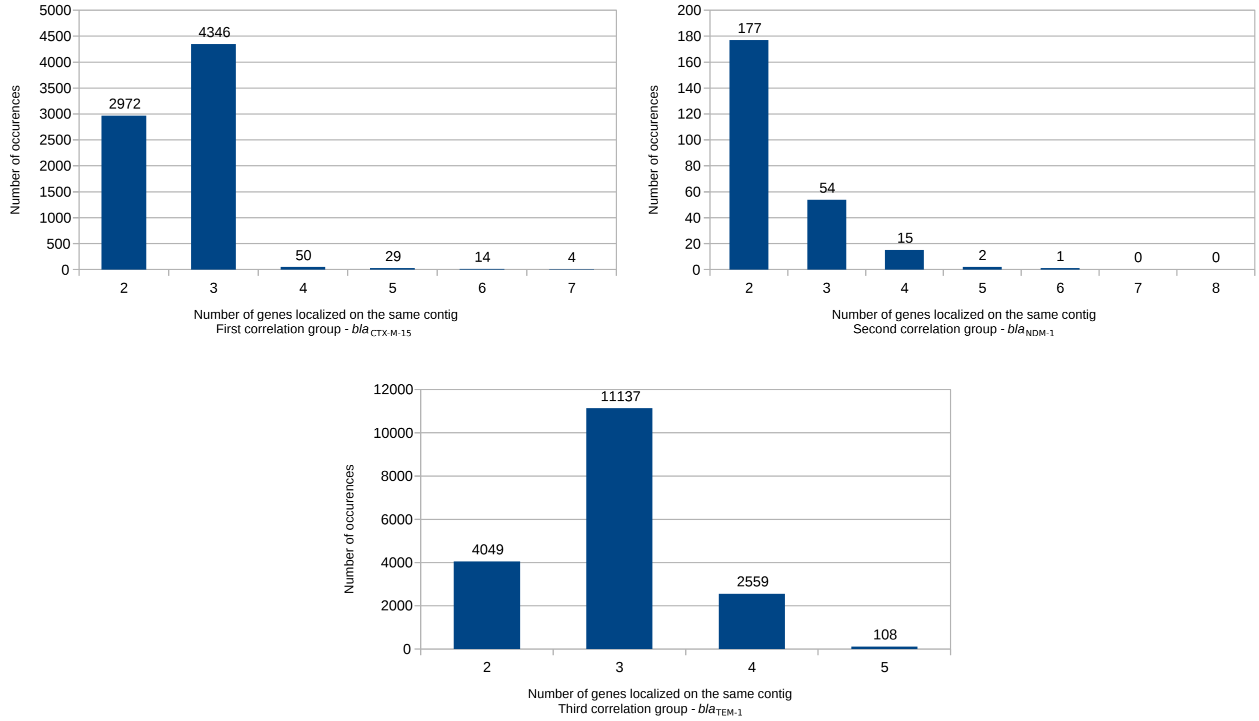

Supplementary Figure 3: Histogram of the number of occurences of colocation of correlated genes. First correlation group corresponds to $bla_{CTX-M-15}$, *aac(6')-Ib, bla*$_{OXA-1}$, *aac(3)-IIa*, *mphA*, *aadA5* and *qacEdelta1* genes. Second correlation group corresponds to bla_{NDM-1}, *aph(3')-VI*, *floR*, *erm-42*, bla_{CMY-6}, *mphE*, *msr(E)* and *armA* genes. Finally, third correlation group correponds to bla_{TEM-1}, *aph(3'')-Ib*, *aph(6)-Id, aac(3)-IId* and *sul2*.

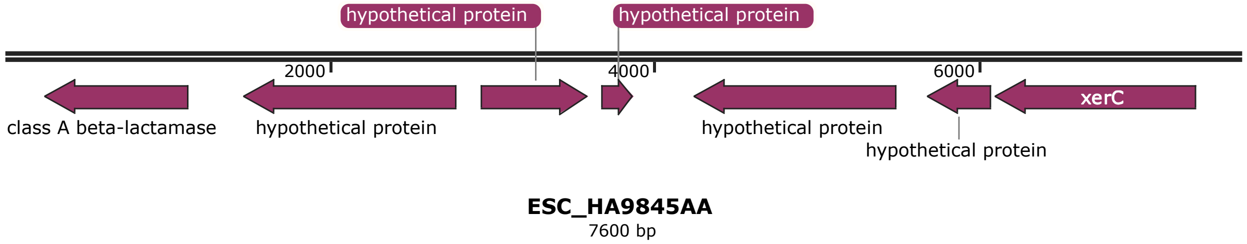

Supplementary Figure 4: Description of the genes shared by the 7,600 bp contig of the strain matching against a *Bacteroides uniformis*. *B. uniformis* genes are coloured in purple.

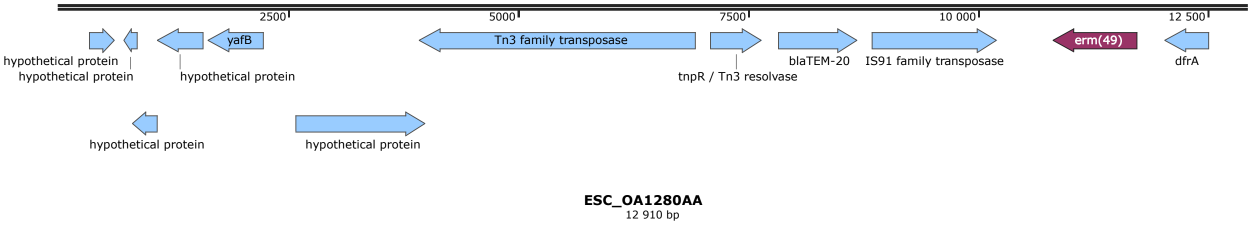

Supplementary Figure 5: Description of the genes shared by the 12,910 bp contig of the strain matching against a *Bifidobacterium breve*. *E. coli* genes are coloured in blue and *B. breve* genes in purple (71 bp in 3' of *erm*(49) also matching with *B. breve* genome).

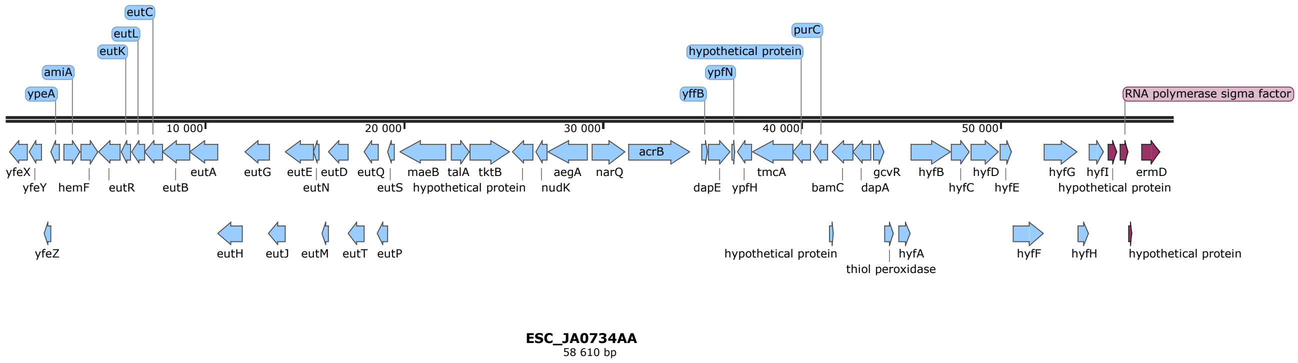

Supplementary Figure 6: Description of the genes shared by the 58,610 bp contig of the strain matching against a *Clostridioides difficile*. *E. coli* genes are coloured in blue and those from *C. difficile* in purple (25 bp in 5' of the first hypothetical protein also matching with *C. difficile).*

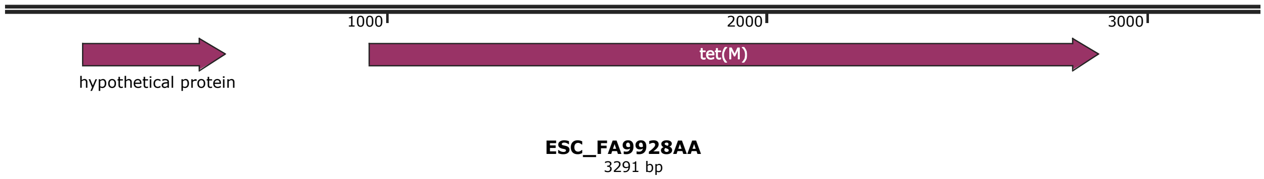

Supplementary Figure 7: Description of the genes shared by the 3,291 bp contig of the strain matching against a *Clostridioides difficile*. C*. difficile* genes are coloured in purple.

Supplementary Table 1: The 27 sub-families of beta-lactamases found in *E. coli*. 100% identity refers to strain containing exactly the sequence of the beta-lactamase and variants correspond to genes sharing at least 80% of identity and/or coverage with those from AMRFinder.

Beta-lactamase sub-family	100 % identity	Variants	Frequency
bla_{TEM}	17,848	516	48.96
bla_{CTX-M}	10,472	168	28.37
$bla_{OXA-1-like}$	4,034	41	10.86
$bla_{CMY-2-like}$	1,938	56	5.32
bla_{SHV}	392	126	1.38
$bla_{OXA-48-like}$	315	0	0.84
bla_{NDM}	296	232	1.41
bla_{KPC}	186	0	0.50
bla_{LAP}	155	6	0.43
bla_{CARB}	138	0	0.37
$bla_{OXA-10-like}$	121	6	0.34
bla_{DHA}	119	21	0.37
bla_{HER}	102	0	0.27
$bla_{OXA-2-like}$	77	0	0.21
$bla_{OXA-PR-like}$	56	0	0.15
bla_{VIM-1}	25	0	0.07
bla_{IMP}	17	0	0.05
bla_{GES}	12	0	0.03
bla_{VEB}	9	0	0.02
bla_{SCO}	8	0	0.02
bla_{ACC}	6	1	0.02
bla_{SFO}	6	0	0.02
$bla_{OXA-5-like}$	2	0	0.01
bla_{FOX}	1	0	0.00
bla_{MOX}	0	1	0.00
bla_{ROB}	0	1	0.00
bla_{SED}	0	1	0.00

Supplementary Table 2: Results of the *glm* function for $bla_{CTX-M-15}$ gene. The first column correspond to the variables (genes, phylogroups and plasmid incompatibility groups), then « Estimate » corresponds to the log of

the odd ratio, « Std. Error » to the standard error, « z value » to the « Estimate » divided by the standard error, « Pr(>|z|) » to the p-value and « Signif » to the significance code (0 '***' 0.001 '**' 0.01 '*' 0.05 '.' 0.1 ' ' 1).

Supplementary Table 3: Results of the *glm* function for *bla*$_{NDM-1}$ gene. The first column correspond to the variables (genes, phylogroups and plasmid incompatibility groups), then « Estimate » corresponds to the log of the odd ratio, « Std. Error » to the standard error, « z value » to the « Estimate » divided by the standard error, « Pr(>|z|) » to the p-value and « Signif » to the significance code (0 '***' 0.001 '**' 0.01 '*' 0.05 '.' 0.1 ' ' 1).

Supplementary Table 4: Results of the *glm* function for *bla*$_{TEM-1}$ gene. The first column correspond to the variables (genes, phylogroups and plasmid incompatibility groups), then « Estimate » corresponds to the log of the odd ratio, « Std. Error » to the standard error, « z value » to the « Estimate » divided by the standard error, « Pr(>|z|) » to the p-value and « Signif » to the significance code (0 '***' 0.001 '**' 0.01 '*' 0.05 '.' 0.1 ' ' 1).

Supplementary Table 5: Genes of Mustard database found in *E. coli*. "100% identity" refers to strain containing exactly the sequence of the genes and "variant" corresponds to variants of the sequence with at least 80% of identity/coverage. AMRFinder Gene ID correspond to ID of gene closely matching to Mustard identified gene. Identity (%) and coverage correspond to value between the AMRFinder and Mustard gene. Complete AMRFinder Gene ID was composed of fields separated by '|' characters and are as follows: Protein GI, RefSeq protein accession, GenBank nucleotide accession, Fusion gene part number (1 if not a fusion gene), Total number of fusion parts (1 if not a fusion gene), Internal family identifier, Internal family class (which is the parent internal family identifier if the protein is an allele, or the internal family identifier otherwise), Resistance mechanism type and Protein name.

Supplementary Table 6: Genes of ResFinderFG database found in *E. coli*. "100% identity" refers to strain containing exactly the sequence of the genes and "variant" corresponds to variants of the sequence with at least 80% of identity/coverage. AMRFinder Gene ID correspond to ID of gene closely matching to ResFinderFG identified gene. Complete AMRFinder Gene ID was composed of fields separated by '|' characters and are as follows: Protein GI, RefSeq protein accession, GenBank nucleotide accession, Fusion gene part number (1 if not a fusion gene), Total number of fusion parts (1 if not a fusion gene), Internal family identifier, Internal family class

(which is the parent internal family identifier if the protein is an allele, or the internal family identifier otherwise), Resistance mechanism type and Protein name.
